# Supplementary material for: Classical Xanthinuria in Nine Israeli Families and Two Isolated Cases from Germany: Molecular, Biochemical and Population Genetics Aspects
Source: Biomedicines. 2021 Jul 7;9(7):788. doi: 10.3390/biomedicines9070788 (PMC8301430; doi:10.3390/biomedicines9070788)
Supplement: Supplementary file 1 [file biomedicines-09-00788-s001.zip › biomedicines-1236880-sm.pdf]

Supporting Information

Historical genealogy of affected families

The families described in this article belong to ancient large clans/tribes with branches living also outside of Israel. Following are results of historical genealogical investigations of the studied families.

Yemenite-Jews

Yemenite-Jews are those Jews who live, or once lived, in Yemen. Families F1, F3 and F8 belong to this ethnic group. Yemenite-Jewish traditions claim that the Jewish settlement in Yemen goes back to biblical times. Archaeological records referring to Judaism in Yemen started to appear during the rule of the South-Arabian Himyarite Kingdom (115–525 CE), whose royal dynasty adopted Judaism in the late 4th century [1]. A large-scale emigration of Yemenite-Jews to Palestine begin in the late 19th century and reached its culmination between 1949 and 1950 after the declaration of the State of Israel. Today the majority of Yemenite-Jews live in Israel. Smaller communities live in the United States and elsewhere and only a handful remained in Yemen. DNA analyses showed that Yemenite-Jews share similar genetic profiles to those of the world's other Jewish communities [2]. A particular example is an ancient Middle-Eastern founder variant c.349G>T (p.(Glu117*)) in the coagulation factor F11 shared by Ashkenazi and non-Ashkenazi Jews, including Yemenite-Jews, and also found among Muslim and Christian-Arabs living in Israel [3,4].

Bedouin-Arab families from the South of Israel (Negev)

The traditional meaning of Bedouin is "desert dweller" or those who live in tents as opposed to sedentary people. However, in Israel this pattern of living of the Bedouins is constantly changing toward a more modern life style.
Family F5 belongs to a large Bedouin tribe in the Negev. Individuals III-5 and III-6 in this family (Figure 1) belong to another clan. The tribe originates from Hijaz (Saudi Arabia), from a coalition of tribes called "Beli". They have family and marriage ties with members of family F7 [39]. Family F7 belongs to large kindred from a tribe with branches both in southern and northern Israel. Genealogically, they come from the tribe of Quraysh from the city Mecca. In Israel they have marriage ties with families F5 and with members of the clan to whom individuals III-5 and III-6 in family **F5** belong [5].
Another Arab family living in the Negev, designated family EL in a former publication [13] belongs to a tribe originating from the city of Gaza. They came to the Negev in the 19th century and married into families in the Negev belonging to several other tribes.

Arab families from the North of Israel

Family F2 belongs to extended Christian-Arab kindred whose members live in several towns and cities of northern Israel. They came here in the 18th century from Lebanon, which was a stop on their route of migration from the region of Mount Huran in Syria [6].
Family F4 belongs to Arab kindred that are originally from the region of Jenin. They have marriage ties with the Bedouin tribes in the north including family F6 [Interview with Ahmad Zakarena, Dayr Ghazala (Jenin), 5 March 2021].
Family F6 belongs to a large Bedouin-Arab tribe, parts of which are concentrated in the region of the Hula and Eastern Galilee and in other regions in northern Israel. Their initial origins are linked to one of the tribes in the southern Arabian Peninsula and Yemen, from where they moved northwards to Iraq, Jordan, Syria, and the region of the Galilee. In Israel this tribe has many marriage ties with other tribes in its locale [41]. The grandmother (III-2) of the second index case (V-2) who introduced the c.913del variant to this family belongs to another Arab-Bedouin tribe living in towns in the Hula Valley and the north of Israel. Some connect it to the wide coalition of those belonging to the tribe of family **F6**, while others claim that the ties are merely marriage ties. But they too probably originate from the southern Arab Peninsula and Yemen and took the same route as members of the tribe to which family F6 belong [7].
Family **F9** belongs to very large kindred from a city located in the Haifa district in the north. The family patriarch came from the Tulkarm District in the mid-17th century. They migrated there in the 13th century from the Jenin District, and to the Jenin District they came from Saudi Arabia. In the macro distribution, this family belongs to the coalition of southern tribes who came from Yemen [8].

Table S1. Clinical data in Families F1, F3 and F8.

Family/case	Sex Age[1]	Genotype	Clinical manifestations							
			UL	Malignancy	Autoimmune	Diabetes	HTN	Dyslip.	NAFLD	CNS
F1 I-1	M 88*	Obl. carrier								
I-2	F 87*	Obl. carrier			Hashimoto		+			
II-1	M 80*	Affected	+		RA/vitiligo	DM1[2]	+			CVA/dementia/tinnitus/leukoaraiosis
II-2	M 78*	Carrier	+			DM1[2]	+	+		CVA/dementia
II-3	M 76*	Carrier		Bladder-TCC		DM1[2]	+	+	+	Dementia/leukoaraiosis
II-4	M 77*	NT		Gallbladder	RA		+	+		Tinnitus/ptosis/microvascular changes
II-5	F 50*	NT		Breast						Schizophrenia
II-6	F 68*	Carrier		Pancreas	Hashimoto	DM1[2]		+		
II-7	M 73	Affected	+	Myeloma	Sjogren's synd.		+		+	
II-8	M 61*	Affected	+	Lung-NSCLC	Overlap synd.	DM1		+		
II-10	F 68	Affected		Breast	Hashimoto					
III-1	F 42	Carrier			Hashimoto					
III-2	M 39	Carrier	+							
F3 II-1	M 72*	Affected				DM2				
F8 III-1	F 85*	Affected		Bladder-TCC colon		DM2				TIA

[1] age in year 2021; * age at death; M–male; F–female; Obl.–obligatory; NT–not tested; + present; UL–urolithiasis; HTN–hypertension; CNS–central nervous system related manifestations; Dyslip.–dyslipidemia; RA–rheumatoid arthritis; Overlap synd.–RA–Sjogren's syndrome–systemic lupus erythematosus–scleroderma–myositis overlap syndrome; DM1–insulin treated diabetes mellitus; DM1[2]–diabetic patients who have been switched from oral hypoglycemic agents to insulin therapy due to inefficacy; DM2–diabetes mellitus type 2; CVA–cerebrovascular accident; TIA–transient ischemic attack; TCC–transitional cell carcinoma of urinary bladder; NSCLC–non-small cell lung cancer;

Table S2. Levels of uric acid (UA), xanthine (X) and hypoxanthine (HX) in 18 affected members of 9 Israeli families and 2 isolated cases from Germany.

Family	Case	UA-S (mg/dl)	UA-U (mg/dl)	X-U	HX-U
F1	II-1	0.1	0	356[1,a]	79 [1,a]
	II-7	-	0	292 [1,a]	192 [1,a]
	II-8	-	0	536 [1,a]	192 [1,a]
	II-10	0	0	455 [1,a]	206 [1,a]
F2	V-2	0	0	106 [3,b]	40 [3,b]
	V-3	0	0	-	-
	V-5	0	0	-	-
F3	II-1	0	0	43.3 [2,a]	23.2 [2,a]
F4	IV-7	0	-	-	-
	V-5	0	0	-	-
	V-6	0	-	-	-
F5	IV-6	0.1	0	-	-
F6	IV-14	0.1	0	-	-
	IV-17	0.1	0	-	-
	V-2	0.1	0	600[3,c]	Traces[3,c]
F7	V-6	0	0	-	-
F8	III-1	0.1	1	-	-
F9	II-3	0	1	-	-
	G1	0.6	0.2	-	-
	G2	0	0.1	-	-

[1] mg/24hrs collection, [2] random sample mg/dl, [3] µmol/mmol creatinine, [a] performed at the Clinical Biochemistry Department, Sackler School of Medicine, Tel Aviv University under the supervision of Prof Esther Shani-Zoref, [b] reported by Dr AH van Gennip from the Academisch Ziekenhuis bij de Universiteit van Amsterdam, Holland, [c] reported by Dr Renate Yakubov, Children's Department, Hilel Yafe Medical Center, Hedera, Israel. See section 2.2 for methods of biochemical tests. Reference ranges: UA-S 2.5-8.0 mg/dl; UA-U 37-92 mg/dl; X-U[1a, 2a] values obtained in three unaffected family members were:19.4, 8 and 6 mg/24hr; HX-U[1a,2a] values obtained in two unaffected family members were 54 and 4.4 mg/24h; X-U[3b] "strongly elevated"; HX-U[3b] " slightly elevated"; \ X-U[3c] 20-40 µmol/mmol creatinine.

Table S3. Homozygosity testing with polymorphic markers within and around the *XDH* gene in families F1, F4 and F6.

Position (bp) on chr.2*	SSR/SNP	F1		F4	F6
		**II-1**	**II-8**	**V-5**	**IV-17**
31,278,307	D2S352[1]	4 7	4 7	-	-
XDH -IVS 30	c.3351-30 A>C[2]	A A	A A	C C	C C
-IVS 29	c.3276+12 G >A[2]	A/G	A/G	G G	G G
-IVS 23	c.2544+35 T>A[2]	A A	A A	A A	T T
31,384,160 *XDH*-IVS 11	D2S2203[1]	4/5	4/5	-	-
XDH-promoter	c.1-337 G>A	-	-	A A	G G
34,26,223	D2S367[1]	7 4	7 4	-	-

*GRCh38.p13 primary assembly, annotation release 109 (https://www.ncbi.nlm.nih.gov/) see full description of SSR/SNP in [1][12], [2] [13], Homozygosity for the rare allele is highlighted by red letters.

Table S4. Allopurinol Loading Test in family **F2.**

Case	V2			
**Time intervals of urine collection (hours)**	**0–6**	**6–12**	**12–18**	**18–24**
Allopurinol*	164	<10	<10	<10
Oxypurinol*	137	276	195	123

* µmol/mmol creatinine, the results have been confirmed by 2-dim. TLC as reported by Dr AH van Gennip from the Academisch Ziekenhuis bij de Universiteit van Amsterdam, Holland.

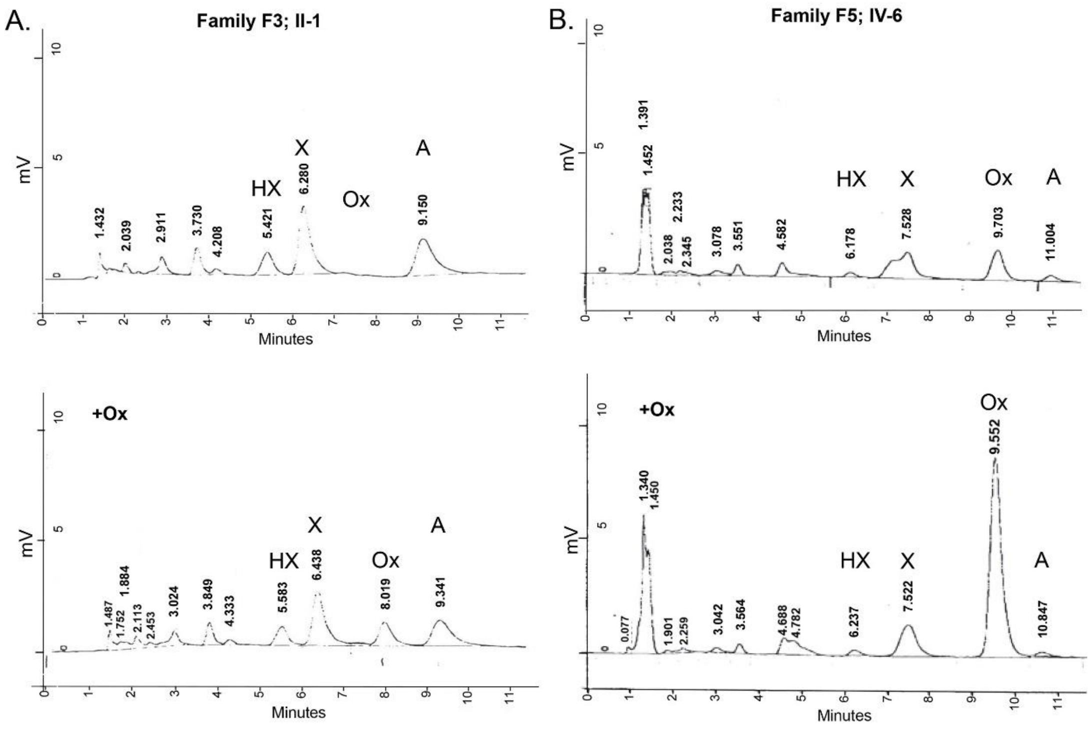

Figure S1. Allopurinol Loading Test in families F3 and F5. HPLC chromatograms of serum samples taken at 180 minutes after administration of allopurinol (**A**) without (upper panels) and with addition (lower panels) of standard oxypurinol (OX) to the serum samples. Note peaks of hypoxanthine (HX), xanthine (X) in all samples and lack of OX peak in family F3 (panel **A**) and its presence in family F5 (panel **B**).

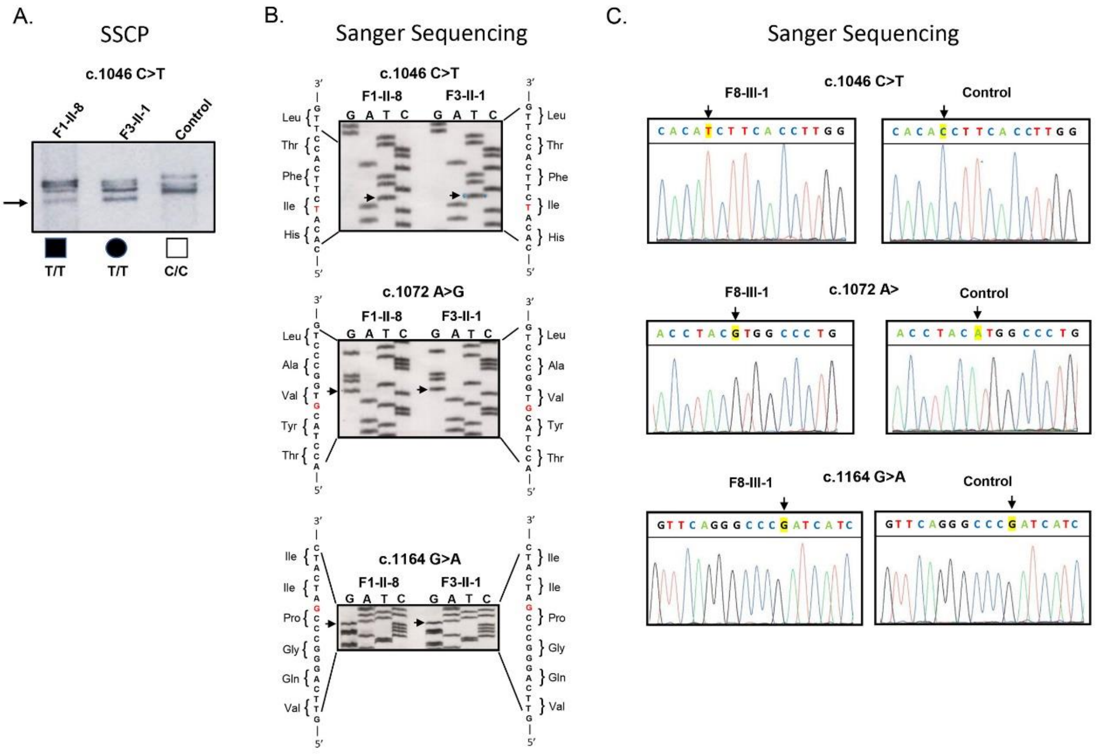

Figure S2. Detection of a c.1046C>T variant in the *MOCOS* gene in families F1, F3 and F8. (**A**) Autoradiogram of an SSCP run of amplified exon 6 of the *MOCOS* gene. Note an aberrant band in samples of affected individuals from families F1 (II-8) and F3 (II-1) as compared to a control sample. (**B**) Autoradiogram of nucleotide sequence analysis of exon 6 of the *MOCOS* gene of affected individuals from families F1 (II-8) and F3 (II-1). Arrows point to the site of the pathogenic variant c.1046C>T and the polymorphic variations c.1072A>G (rs678560) and c.1164 G>A (rs667667). (**C**) Chromatogram of nucleotide sequence analysis of exon 6 of the *MOCOS* gene of the affected individual III-1 from family F8 and a control sample. Arrows point to the pathogenic variant c.1046C>T and the polymorphic variants c.1072A>G and c.1164 G>A. Note the identical homozygous genotypes of the affected individuals from all three families at the polymorphic and pathogenic variants' sites (5'-TGG).

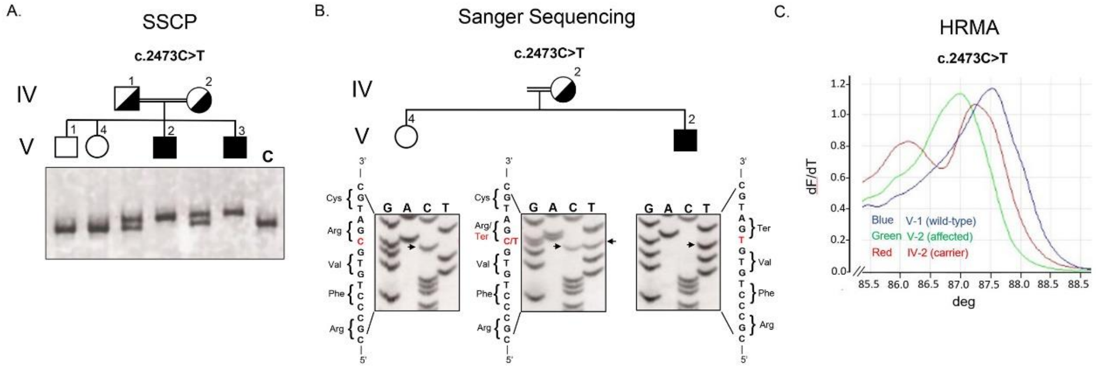

Figure S3. Detection of the c.2473C>T variant in the *XDH* gene in family F2 and a specific HRMA assay. (**A**) Autoradiogram of an SSCP run of amplified exon 23 of the *XDH* gene in 6 members of family F2 and a control sample (**C**) Note the separation of specific bands of the wild-type and variant alleles. (**B**) Autoradiogram of nucleotide sequence analysis of exon 23 of the *XDH* gene in a wild-type (V-4), a carrier (IV-2) and an affected (V-2) member of family F2. The arrows point to the variant nucleotide c.2473 C>T. **C.** HRMA assay showing differential melting curves (delta fluorescence/delta time) in DNA samples of wild-type (blue), carrier (red) and affected (green) family members. Note the allele specific temperatures of melting.

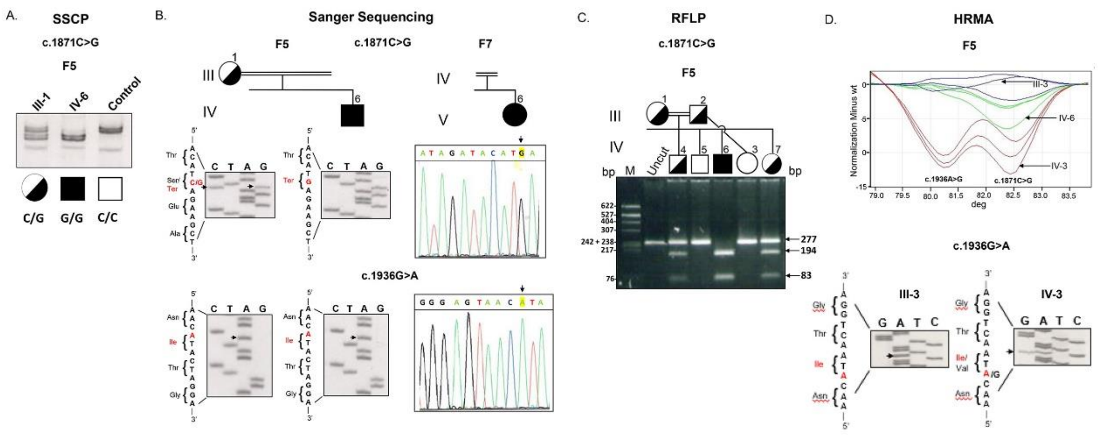

Figure S4. Detection of a c.1871 C>G variant in the *XDH* gene in families F5, and F7. (**A**) Autoradiogram of an SSCP run of amplified exon 18 of the *XDH* gene in a carrier (III-1) and a homozygous affected (IV-6) member of family F5 and two control samples (C). Note the separation of bands specific to the wild-type and variant alleles. (**B**) Autoradiogram of nucleotide sequence analysis of exon 18 of the *XDH* gene in a carrier (III-1) and a homozygous affected (IV-6) member of family F5 and chromatogram of nucleotide sequence analysis of a homozygous affected individual (V-6) of family F7. The arrows point to the pathogenic c.1871C>G and polymorphic c.1936A<G variants' sites. Note that all 3 samples shown in this panel are homozygous for the common A allele at the polymorphic c.1936A>G site. (**C**) RFLP assay showing recognition of the pathogenic variant c.1871C>G allele by the Nla III restriction enzyme (fragments of 194 and 83 bp) as opposed to the wild type allele that is uncut. (**D**) HRMA assay showing the melting profile of samples of homozygous wild-type (III-3, blue curve), homozygous affected (IV-6, green curve) and heterozygous carrier (IV-3, red curve) of the c.1871C>G variant in family F5. Note in the heterozygous carrier sample IV-3 an additional melting point at 80.25 °C due to heterozygosity at the polymorphic c.1936 A>G site as can be seen in the attached autoradiogram of the nucleotide sequence analysis.

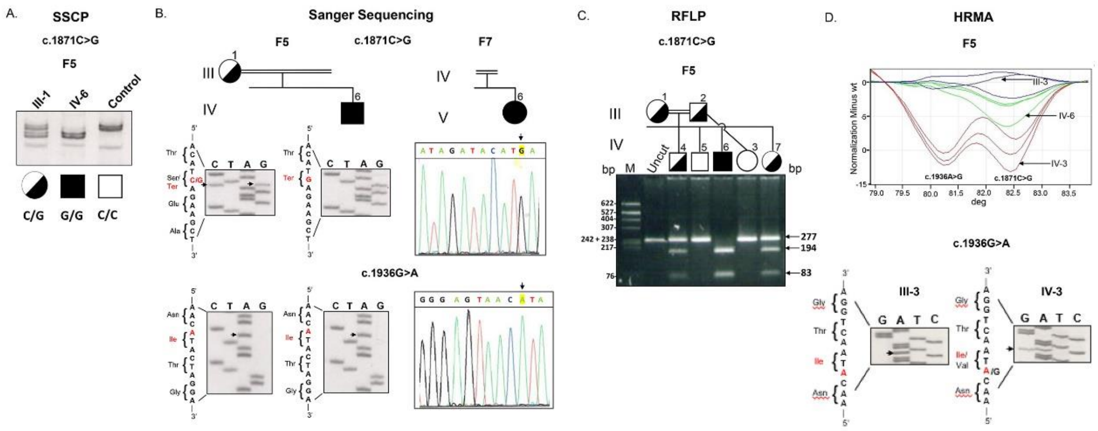

Figure S5. Detection of the c.141insG and c.913delC variants in in exons 3 and 11, respectively, of the *XDH* gene in Figure 6. (**A**) Autoradiogram of an SSCP run of amplified exon 3 of the *XDH* gene in 5 samples. From left to right: wild-type control (C1), homozygous variant (IV-15), homozygous wild-type (IV-16) and two heterozygous carriers (III-5, III-6) of the c.141insG variant. In the lower part of the autoradiogram it can be seen that the run of the homozygous variant (c.141insG) does not differ from the run of the control (C1) and homozygous wild type (IV-16) samples, however an extra heteroduplex band can be seen in the heterozygous (III-5, III-6) samples (arrow at the right). On the upper part of the autoradiogram the SSCP pattern reflects the c.102-35 C>G polymorphism in IVS 2 of the XDH gene within the amplified genomic fragment (see results of sequence in the lower part of panel **B**). (**B**). Autoradiogram of nucleotide sequence analysis of exon 3 of the *XDH* gene in homozygous affected (IV-15), two heterozygous carriers (III-5, III-6) of the c.141insG variant and a control sample (C2). The upper panel shows the sequence around the pathogenic variant and the lower panel shows the sequence around the polymorphic c.102-35 C>G site and chromatograms of nucleotide sequence analysis of a compound heterozygous (c.141insG/c.913delC) affected individual (V-1). (**C**) RFLP assay showing abolishment of an Mwo I restriction site by the c.913delC variant. (**D**) HRMA assays developed for detection of the c.141insG and c.913delC variants.

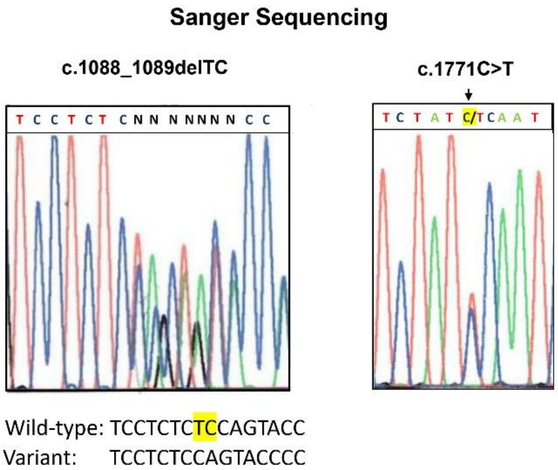

Figure S6. Detection of the c.1088_1089delTC and c.1771C>T variants in the *MOCOS* gene in case G1. Chromatograms of nucleotide sequence analysis showing the c.1088_1089delTC and c.1771C>T variants identified in case G1.

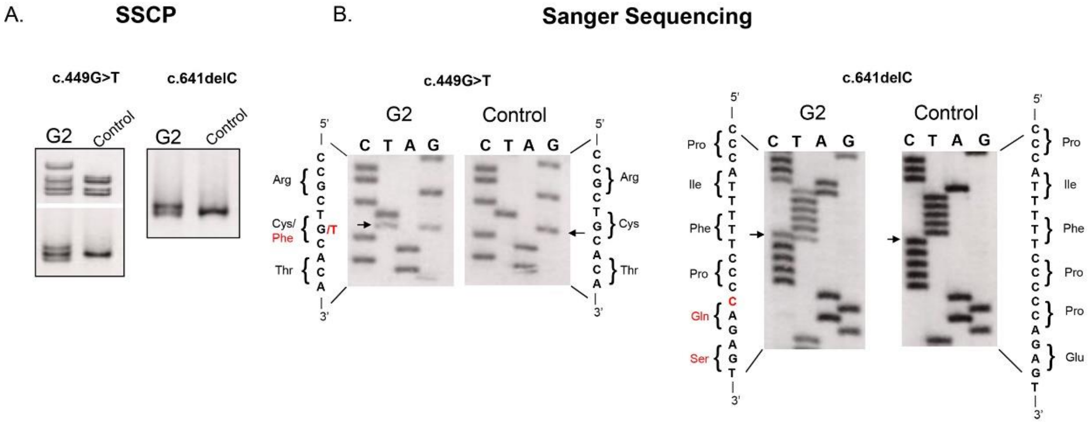

Figure S7. Detection of the c.449G>T and c.641delC variants in the *XDH* gene in case G2. (**A**) Autoradiograms of SSCP runs of amplified exons 6 and 8 of the *XDH* gene in case G2 and a wild-type control. (**B**) Autoradiograms of nucleotide sequence analysis of exons 6 and 8 of the *XDH* gene in case G2 and a wild-type control.

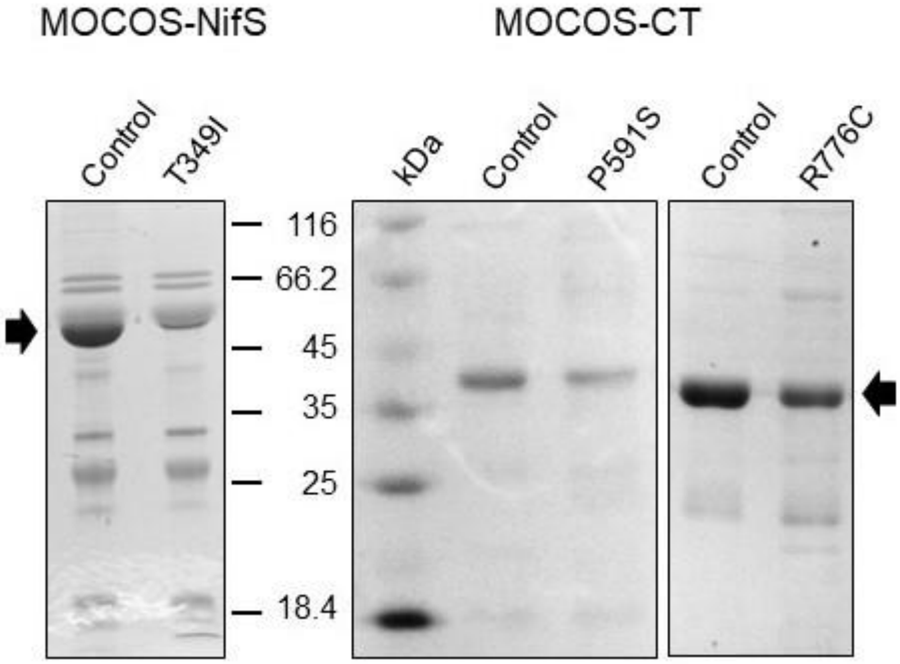

Figure S8. Analysis of recombinant human MOCOS domains expressed in *E. coli* and purified via Ni-NTA affinity chromatography. Left panel: 15 µg of recombinant MOCOS-NifS proteins were electrophoresed on 12 % SDS polyacrylamide gels and staining with Coomassie Brilliant Blue. MOCOS-NifS proteins present a molecular mass of 56 kDa as indicated by a black arrow at the left side. Middle and right panel: 3 µg and 6 µg, respectively, of recombinant MOCOS-CT proteins were electrophoresed on 12 % SDS polyacrylamide gels and staining with Coomassie Brilliant Blue. MOCOS-CT proteins present a molecular mass of about 38 kDa as indicated by a black arrow at the right side. Note the difference in the intensity of the variant vs the control NifS (56 kDa) and MOCOS-CT (38 kDa) protein bands. Of note, the yield of the MOCOS-NifS WT is up to 8 mg protein/L expression culture, whereas the yield of MOCOS-NifS T349I is 0.7 mg protein/L expression culture (of the shown single preparation, others are in the same range but with less T349I protein of 56 kDa relative to proteins of other molecular mass).

References

1. Eraqi Klorman, B-Z. The Jews of Yemen. https://www.oxfordbibliographies.com/view/document/obo-9780199840731/obo-9780199840731-0168.xml LAST MODIFIED: 26 APRIL 2018, retrieved march 17 2021.
2. Hammer, M.F.; Redd, A.J.; Wood, E.T.; Bonner, M.R.; Jarjanazi, H.; Karafet, T.; Santachiara-Benerecetti, S.; Oppenheim, A.; Jobling, M.A.; Jenkins, T.; Ostrer, H.; Bonne-Tamir, B. Jewish and Middle Eastern non-Jewish populations share a common pool of Y-chromosome biallelic haplotypes. *Proc. Natl. Acad. Sci.* **2000**, *97*, 6769–6774, doi:10.1073/pnas.100115997.
3. 3. Peretz, H.; Mulai, A.; Usher, S.; Zivelin, A.; Segal, A.; Weisman, Z.; Mittelman, M.; Lupo, H.; Lanir, N.; Brenner, B.; Shpilberg, O.; Seligsohn, U. The two common mutations causing factor XI deficiency in Jews stem from distinct founders: One of ancient Middle Eastern origin and another of more recent European origin. *Blood* **1997**, *90*, 2654–2659.
4. Goldstein, D.B.; Reich, D.E.; Bradman, N.; Usher, S.; Seligsohn, U.; Peretz, H. Age estimates of two common mutations causing factor XI deficiency: Recent drift is not necessary for elevated disease incidence among Ashkenazi Jews. *Am. J. Hum. Genet.* **1999**, *64*, 1071–1075, doi:10.1086/302313.
5. Kabha, M. The oral history of the Arab population in the Negev. Center for Studies of the Bedouin Society, Ben Gurion University of the Negev, **2011**.
6. al-Dabagh, M.M. al-Qaba'il al-'Arabiyya wa-sulalatha fi Filastin (The Arab tribes and their descendants in Palestine), Dar al-Yasar, Baqa al-Gharbiyya, **1988**. (in Arabic).
7. 'Araaf, S. Bidu Marj bin ʿAmer wa-al-Jaliliyin (The Bedouin in the Jezreel Valley and the Upper and Lower Galilee). Center for Rural Studies, Nazareth, **2001**. (in Arabic).
8. Bayadsa, S. Baqa al-Gharbiyya, Tarikh la yunsa (Baqa al-Gharbiyya unforgettable history). Dar al-Hoda, Kufr Qarʿa, **2002**, pp. 133-135. (in Arabic).
